# Sleep and eyewitness memory: Fewer false identifications after sleep when the target is absent from the lineup

**DOI:** 10.1371/journal.pone.0182907

**Published:** 2017-09-06

**Authors:** Michelle E. Stepan, Taylor M. Dehnke, Kimberly M. Fenn

**Affiliations:** Department of Psychology, Michigan State University, East Lansing, MI, United States of America; Waseda University, JAPAN

## Abstract

Inaccurate eyewitness identifications are the leading cause of known false convictions in the United States. Moreover, improving eyewitness memory is difficult and often unsuccessful. Sleep consistently strengthens and protects memory from interference, particularly when a recall test is used. However, the effect of sleep on recognition memory is more equivocal. Eyewitness identification tests are often recognition based, thus leaving open the question of how sleep affects recognition performance in an eyewitness context. In the current study, we investigated the effect of sleep on eyewitness memory. Participants watched a video of a mock-crime and attempted to identify the perpetrator from a simultaneous lineup after a 12-hour retention interval that either spanned a waking day or night of sleep. In Experiment 1, we used a target-present lineup and, in Experiment 2, we used a target-absent lineup in order to investigate correct and false identifications, respectively. Sleep reduced false identifications in the target-absent lineup (Experiment 2) but had no effect on correct identifications in the target-present lineup (Experiment 1). These results are discussed with respect to memory strength and decision making strategies.

## Introduction

Several decades of research have shown that sleep is not only beneficial for physiological function but is also important for memory. Sleep strengthens hippocampal-dependent declarative memory for word pairs [1–4] and spatial information [5–7]. Sleep also protects memory from interference experienced during waking [8–9], likely due to an active consolidation process [10–13, 3–4, 6–7].

Although a vast literature supports the positive influence of sleep on memory, the effect of sleep on eyewitness memory remains unexplored. Moreover, it is difficult to predict the effect of sleep on eyewitness memory due to the reliance on free or cued recall tests in the sleep-dependent consolidation literature and a comparable dearth of work using recognition tests. During free recall, participants recall a list of previously studied items, whereas in cued recall, participants are given an item from a set of studied items in order to aid, or cue, recall of a particular item. In contrast, recognition tests differ from recall in that individual items are presented to a participant who must then indicate if the item had been previously studied. It is well-established that sleep strengthens veridical memory when recall tests are used [1–5, 7–9], but few studies have used recognition tests (the type of test used in eyewitness identification research).

Studies directly testing the effect of sleep on recognition memory have been equivocal. Recognition of emotionally salient information is better after sleep [13–14] and recognition of neutral information is enhanced after a daytime nap [15–16]. Studies testing the effect of a full night of sleep on neutral information have used the Deese-Roediger-McDermott (DRM) illusory memory paradigm [17–18]. These studies found no effect of sleep on correct recognition of neutral words but show either a reduction in false recognition following sleep [19–20] or no effect of sleep on false recognition [21]. It should be noted that the effect of sleep on false memory seems to be sensitive to testing format as the reverse effect (higher false memory after sleep) is found when a recall test is used [22–24]. The DRM paradigm, however, is unique in that recognizing one item can improve recognition for other items in the list, which may explain the null result for correct recognition. Thus, it is unclear whether the null effect of sleep on correct recognition is restricted to the DRM paradigm or if this null result accurately reflects the effect of sleep on recognition for neutral information across paradigms.

We investigated the effect of sleep on recognition in an eyewitness memory paradigm to help disambiguate prior results and to broaden the research on sleep-dependent memory consolidation to an ecologically important stimulus. Participants watched a video of a mock-crime and attempted to identify the perpetrator after a retention interval that either spanned a waking day or night of sleep. Experiment 1 utilized a lineup in which the perpetrator was present in the lineup (target-present); Experiment 2 utilized a lineup in which the perpetrator was not present (target-absent). Given substantial findings that sleep is beneficial for memory, we predicted that sleep would facilitate memory retention, resulting in more correct identifications in the target-present lineup, and protect against false recognition, resulting in fewer false identifications in the target-absent lineup.

## Experiment 1

### Methods

#### Participants

This study was approved by the Michigan State University Institutional Review Board and meets the federal guidelines for human subjects. We recruited undergraduate students 18–25 years old from Michigan State University with no sleep or memory disorders and who had not seen the 60 Minutes special on eyewitness memory [25]. Participants were enrolled in an introductory psychology course that had not discussed eyewitness memory and received course credit. After exclusions (See S1 Supporting Information), we obtained a sample of 198 participants (138 females, *M*_age_ = 18.90, *SD* = 1.14).

#### Materials

Mock-crime video. The mock-crime video participants watched was modeled after a rooftop bomber video that was used successfully in prior research [26]. The 13.3-second video was shot from the perspective of a witness viewing a young white male planting a bomb on a rooftop. He looks directly at the camera twice before leaving the premises.

Recognition lineup test. Participants were tested using a simultaneous (suspects all shown together) target-present (Experiment 1) or target-absent (Experiment 2) lineup consisting of six headshots centered in a row in grayscale. Target-present lineups consisted of the perpetrator and a random selection of five out of six possible fillers who were found to look similar to the perpetrator in pilot testing. Target-absent lineups contained all six fillers but not the perpetrator. The location of each picture was randomized so that the perpetrator and fillers could appear in any position within the lineup. Numbers 1 through 6 appeared below each photo and participants were instructed to press the number to identify that individual as the perpetrator or to press the spacebar to indicate that the perpetrator was absent.

Stanford Sleepiness Scale (SSS). SSS [27] assesses how tired or alert participants feel on a 7-point Likert scale (from “Feeling active, vital, alert, or wide awake” to “No longer fighting sleep, sleep onset soon, having dream-like thoughts” with higher numbers corresponding to greater sleepiness.

Positive and Negative Affect Schedule (PANAS). PANAS [28] assesses positive and negative affect. Participants rate 20 adjectives on how well each item describes their current mood ranging from “very slightly or not at all” to “extremely.”

Operation Span (OSPAN). OSPAN [29] was used to assess working memory capacity. For each trial, participants verified whether an answer provided to an arithmetic problem was true or false and were shown a letter to remember. Each string contained between three and seven math problems and letters. After each string, participants recalled the letters using a 3X4 grid that had a letter presented in each square of the grid for a total of 12 letter options. Participants received three of each string length for a total of 15 strings or 75 letters.

Morningness Eveningness Questionnaire (MEQ). MEQ [30] assesses time-of-day preference using a 19-item scale. Based on the score, participants are categorized as either “Definite Morning,” “Moderate Morning,” “Definite Evening,” “Moderate Evening,” or “Neither Morning nor Evening.”

Pittsburgh Sleep Quality Index (PSQI). PSQI [31] measures overall sleep quality with questions in seven key areas: subjective sleep quality, sleep latency, sleep duration, habitual sleep efficiency, sleep disturbances, use of sleep medication, and daytime dysfunction.

Sleep diaries. For approximately one week prior to the experiment, participants recorded their sleep, including the time they went to bed and woke up and the number and duration of awakenings during the night.

#### Procedure

All participants completed two phases: Encoding and Test. For experimental participants, the two phases were separated by a 12-hour retention interval. The Wake group (n = 47) completed Encoding in the morning (08:00–10:00) and Test in the evening (20:00–22:00). The Sleep group (n = 41) completed Encoding in the evening (20:00–22:00) and Test the following morning (08:00–10:00). Control participants completed both phases in one session, either in the morning, 08:00–10:00 (AM, n = 51), or evening, 20:00–22:00 (PM, n = 59) and were used to assess diurnal or circadian effects on performance. Control participants completed both phases in a single session since this design controls for time-of-day effects without introducing the confound of awake time after encoding, which is known to influence memory [3].

At the start of the Encoding phase, informed written consent was obtained from all participants. Participants then completed sleepiness (SSS) and mood (PANAS) assessments and watched a single mock-crime video. Participants were instructed to pay close attention to the video. Although multiple observations per participant would be preferable in eyewitness memory research, this would not be a feasible design given the 12-hour delay between the Encoding and Test phases. We chose to use a single observation per participant in order to avoid interference effects caused by watching multiple mock-crime videos in a row and to prevent confusion if multiple tests were given in succession. Following the video, participants completed OSPAN to reduce rehearsal. This phase took approximately 30 minutes.

The Test phase consisted of SSS, PANAS, and the target-present recognition lineup test. Before viewing the lineup, we gave participants unbiased instructions highlighting the fact that the perpetrator may not be in the lineup, “Look at each man closely and decide if he was the man who committed the crime. Please be aware that the culprit may or may not be present in the lineup.” After making a response, participants rated their confidence on a 7-point Likert scale and completed the MEQ, PSQI, and a demographic questionnaire. This phase took approximately 20 minutes.

### Results

#### Memory performance

Recognition lineup test. Responses were coded into one of three possible categories: Correct Identification (successful identification of the perpetrator), False Identification (incorrect identification of an innocent filler), or No Identification (indication that the perpetrator was not present). We compared performance between the Wake and Sleep groups using a Chi-Square test. We predicted that Sleep would show a higher percentage of Correct Identifications but the groups did not differ, χ^2^(2, *N =* 88) = .43, *p =* .81 (Table 1).

**Table 1:** Recognition lineup test performance and confidence scores in Experiment 1.

	Lineup Performance			Confidence		
	Correct ID	False ID	No ID	Correct ID	False ID	No ID
**Wake**	51% (24)	23% (11)	26% (12)	4.79 (1.10)	3.00 (1.48)	4.50 (1.00)
**Sleep**	49% (20)	29% (12)	22% (9)	5.15 (.93)	4.08 (1.00)	3.89 (1.62)
**AM**	67% (34)	12% (6)	22% (11)	4.97 (1.11)	4.00 (1.79)	4.36 (1.43)
**PM**	63% (37)	15% (9)	22% (13)	5.14 (1.38)	3.56 (1.24)	4.69 (1.75)

Under lineup performance, the percentage and total number of participants (in parenthesis) who made a Correct, False, or No Identification in the experimental (Wake, Sleep) and control groups (AM, PM) in Experiment 1. Under confidence, the mean reported confidence and standard deviations (in parenthesis) for Correct, False, and No Identifications in the experimental and control groups. Higher numbers indicate more confidence.

Next, we compared confidence using a two factor between-subjects ANOVA with Identification category and Condition as factors. There was a significant effect of Identification category, *F*(2, 82) = 12.00, *MSE =* 1.33, *p* < .001. Post-hoc Tukey tests confirmed that confidence for Correct Identifications was higher than for False Identifications but not higher than No Identifications. Confidence for False and No Identifications did not differ. There was no main effect of Condition, *F*(1, 82) = 1.12, *MSE =* 1.33, *p =* .29, and no interaction, *F*(2, 82) = 2.93, *MSE =* 1.33, *p =* .06 (Table 1).

To ensure that there were no diurnal or circadian effects, we compared performance between the AM and PM control groups. As expected, we found no difference in ability to recognize the perpetrator, χ^2^(2, *N* = 110) = .31, *p =* .86 (Table 1). We also compared confidence using a between-subjects ANOVA with Identification category and Time (AM, PM) as factors. There was a significant main effect of Identification, *F*(2, 104) = 5.67, *MSE =* 1.87, *p =* .01; confidence was higher for Correct than False Identifications but not reliably different from No Identifications, based on post-hoc Tukey tests. There was no main effect of Time and no interaction, *F’s* < 0.5, *p’s >* .69 (Table 1).

#### Control measures

Sleepiness and mood. We found group differences between the Wake and Sleep groups in sleepiness and mood. However, follow-up analyses confirmed that neither sleepiness nor mood were significant predictors of performance during the lineup test (see S1 Supporting Information for full analyses).

Other control measures. We also assessed working memory capacity, chronotype, sleep quality, and sleep duration prior to the experiment and found no significant differences between Wake and Sleep or between AM and PM for any measure (See S1 Supporting Information for full analyses).

## Experiment 2

Although the Wake and Sleep groups showed similar ability to correctly identify the perpetrator, sleep may still affect performance in a target-absent lineup. Previous studies found lower false recognition after sleep, despite no effect on correct recognition. Therefore, we next used a target-absent lineup to gain a more complete understanding of the effect of sleep on eyewitness memory. We predicted that there would be fewer False Identifications after a retention interval that included sleep compared to wakefulness.

### Methods

#### Participants

Recruitment used the same criteria from Experiment 1. After exclusions (S1 Supporting Information), we attained a sample of 238 participants (174 females, *M*_age =_ 18.89, *SD =* 1.10).

#### Procedure

The materials and procedure were the same as Experiment 1 except that we used a target-absent lineup.

### Results

#### Memory performance

Recognition lineup test. The perpetrator was not in the lineup, so there were two possible response categories: False and No Identifications. We used a Chi-Square test to determine if performance differed between Wake (n = 53) and Sleep (n = 43). As predicted, the Sleep group made fewer False Identifications than Wake, χ^2^(1, *N* = 96) = 5.61, *p =* .02 (Table 2).

**Table 2:** Recognition lineup test performance and confidence scores in Experiment 2.

	Lineup Performance		Confidence	
	False ID	No ID	False ID	No ID
**Wake**	66% (35)	34% (18)	3.69 (1.11)	4.56 (1.46)
**Sleep**	42% (18)	58% (25)	3.67 (1.46)	4.60 (.91)
**AM**	43% (31)	58% (42)	4.06 (1.18)	4.81 (1.33)
**PM**	41% (28)	59% (41)	4.36 (1.57)	4.54 (1.47)

Under lineup performance, the percentage and total number of participants (in parenthesis) who made a False or No Identification in the experimental (Wake, Sleep) and control groups (AM, PM) in Experiment 2. Under confidence, the mean reported confidence and standard deviations (in parenthesis) for False and No Identifications in the experimental and control groups. Higher numbers indicate more confidence.

To follow-up on the significant effect of sleep on the lineup test, we conducted an additional analysis to help determine the underlying cause. Although prior literature has established that sleep plays an important role in the consolidation of declarative memories [11, 32–33], we cannot entirely rule out that the current results were driven by a passive protection from interference. Therefore, we examined whether time spent awake after encoding (i.e. amount of interference) in the Sleep group was related to lineup test performance. For one week prior to the study, we collected information on participants’ habitual bedtime. The average habitual bedtime for the Sleep group was fifty-three minutes after midnight, meaning that participants in this group remained awake for an average 3 hours and 39 minutes after encoding. A logistic regression showed that estimated time awake after encoding in the Sleep group was not related to lineup test performance (β = -.01, p = .30), as one would predict it would have been from an interference account of the current results. This suggests that sleep may have actively consolidated memory for the perpetrator.

Next, we compared confidence using a two factor between-subjects ANOVA with Condition and Identification category (False, No Identification) as factors. There was a significant main effect of Identification, *F*(1, 92) = 12.43, *MSE =* 1.46, *p =* .001, with higher confidence for No Identifications than False Identifications. The main effect of Condition and the interaction were not significant, *F’s* < 0.02, *p’s >* .90 (Table 2).

We also compared performance between the AM (n = 73) and PM (n = 69) control groups and found that lineup performance, χ^2^(1, *N* = 142) = .05, *p =* .82, and confidence, *F*(1, 138) = .002, *MSE =* 1.94, *p =* .97, were similar between groups. Once again, participants reported higher confidence for a No identification than a False identification, *F*(1, 138) = 3.70, *MSE =* 1.94, *p =* .05. The interaction was not significant, *F*(1, 138) = 1.42, *MSE =* 1.94, *p =* .24 (Table 2).

#### Control measures

Sleepiness and mood. There were group differences for sleepiness and mood but follow-up analyses revealed that neither sleepiness nor mood predicted performance (see S1 Supporting Information for full analyses).

Other control measures. There were no significant differences between Wake and Sleep or AM and PM control groups for working memory capacity, chronotype, sleep quality, or average sleep duration (See S1 Supporting Information for full analyses).

## General discussion

The current study extends the literature on sleep-dependent memory consolidation by integrating two disparate fields of inquiry–sleep-dependent memory consolidation and eyewitness memory. Participants watched a video of a mock-crime and attempted to identify the perpetrator from a simultaneous lineup after a 12-hour retention interval that either spanned a waking day or a night of sleep. When the perpetrator was absent from the lineup, participants were more likely to correctly reject all fillers if tested after a period of sleep compared to wakefulness. However, when the perpetrator was in the lineup, sleep did not affect the ability to correctly identify the perpetrator. These results cannot be explained by circadian or diurnal effects because control participants performed similarly in both the target-present and target-absent lineups, regardless of time-of-day. The present results also cannot be explained by floor performance masking effects that otherwise would have been detected. A meta-analysis found that participants made a false identification in target-absent simultaneous lineups 51% of the time and correct identifications of the perpetrator 50% of the time in target-present simultaneous lineups [34]. Consistent with prior research, control participants made a False Identification in the target-absent lineup 42% of the time and experimental participants made a Correct Identification 50% of the time. Lastly, although we found differences in sleepiness and mood between the groups, regression analyses indicate that neither sleepiness nor mood predicted performance during the lineup test.

The present results also help to disambiguate equivocal findings in the literature regarding the role of sleep on recognition memory, especially correct recognition. Some studies using emotional stimuli show higher correct recognition after sleep [13–14]. However, our pattern of results was consistent with recognition studies using the DRM paradigm which find that sleep protects against false recognition but does not affect correct recognition [19–20]. Our eyewitness memory paradigm is quite different from the DRM paradigm, which suggests that this pattern of results generalizes and may accurately reflect recognition memory performance of neutral information following sleep. Although witnessing an actual crime can be a highly emotional experience, the mock-crime video used in this experiment was not designed to be emotionally arousing. Thus, the relationship between sleep and correct recognition performance may be dependent on the emotional nature of the stimuli.

Although our results align with previous research on recognition memory, it is unclear why sleep produces this pattern of results. These results could be explained either by an active consolidation process [11] or through passive protection from interference [35]. Although we did not find a difference in correct identifications in the target-present lineup, a long line of research has shown that sleep is beneficial for declarative memory (for reviews see [11, 32–33]). Sleep may have actively strengthened memory in our experiments, but our single-trial measure may not have been sensitive enough to detect small differences in memory strength. However, memory strength may have had an indirect effect on performance through decision making strategies, an important component of eyewitness identification tests [36]. For example, absolute strategies (comparing each suspect to memory) and relative strategies (comparing suspects to each other to determine who most resembles the perpetrator *relative* to the others) are both useful in positively identifying the perpetrator when he is in the lineup. However, relative strategies tend to increase false identifications when the perpetrator is absent from the lineup, largely because they ignore the possibility that the perpetrator might not be in the lineup [37–38]. Both strategies are used in simultaneous lineups [39–41]. Individuals likely need strong or easily accessible memories to directly compare suspects to memory and implement an absolute strategy. Those with weak memory may be more likely to show signs of deliberation (e.g. revisiting or comparing lineup members) [40], indicative of a relative strategy. Thus, it is possible that sleep strengthened memory and increased the likelihood of using an absolute strategy, which then more directly affected lineup identifications. This could explain the decrease in False Identifications in the target-absent lineup without a corresponding increase in Correct Identification.

In contrast, these results could also be explained by passive protection from interference during sleep. In this account, sleep does not strengthen memory but provides a period of time in which the memory for the perpetrator was protected from the damaging effects of interference. In contrast, participants in the Wake group remained awake for 12 hours–likely encountering other faces that may have interfered with their memory for the perpetrator. Although it is possible that interference would decrease performance in both target-present and target-absent lineups, the presence of the actual perpetrator in the target-present lineup may have triggered enough familiarity that participants in the Wake group were able to frequently make a Correct Identification. Therefore, the target-absent lineup may be more sensitive to detecting small changes in memory strength between the groups.

Although consolidation during sleep or passive protection from interference could account for the pattern of results in the current study, the passive interference account falls short in several regards. First, while the Wake group undoubtedly experienced more interference overall, both the Wake and Sleep groups experienced interference at the most critical time for new memories–immediately after encoding [42–43]. Both groups completed Operation Span, an engaging task that takes approximately 20 minutes to complete, immediately after watching the mock-crime video. Additionally, we estimate that participants in the Sleep group spent an additional 3 hours and 39 minutes awake after encoding, time in which they likely encoded much interfering information. Despite this, the Sleep group made fewer False Identifications in a target-absent lineup. Second, estimated time spent awake after encoding in the Sleep group was not related to lineup performance. If amount of interference experienced during waking hours was driving the current result for the target-absent lineup, then individuals who spent less time awake should show better lineup performance. Therefore, while we do not entirely rule out interference as a factor influencing the current results, a purely interference-based account appears insufficient to fully explain the current findings.

Using an eyewitness design, this study provides additional insight on sleep and recognition memory. Consistent with previous research using neutral stimuli, we found that sleep reduced false identifications in a target-absent lineup but had no effect on correct identifications in a target-present lineup. These results could reflect changes in memory strength and decision making strategies after sleep, or they could be the result of increased interference after waking. Further research is needed to fully understand how sleep affects eyewitness memory, recognition memory, and decision making. However, the current study provides an important first step towards understanding how the complicated systems of sleep-dependent consolidation, memory, and decision making interact to influence behavior.

## Supporting information

S1 Supporting Information Additional methods and analyses of control measures. (DOCX)
