# Supplementary material for: Sleep and eyewitness memory: Fewer false identifications after sleep when the target is absent from the lineup
Source: PLoS One. 2017 Sep 6;12(9):e0182907. doi: 10.1371/journal.pone.0182907 (PMC5587105; doi:10.1371/journal.pone.0182907)
Supplement: S1 Supporting Information — (DOCX) [file pone.0182907.s001.docx]

**Sleep and eyewitness memory:**

**Fewer false identifications after sleep when the target is absent from the lineup**

Michelle E. Stepan, Taylor M. Dehnke, Kimberly M. Fenn

**Supporting Information**

**Experiment 1**

**Additional Methods**

**Participants.** We excluded participants for: incomplete datasets (n = 19), previously watching the 60 Minutes special (n = 9), sleeping less than 4 hours the night before the Encoding or Test phase (n = 7), reporting a sleep disorder (n = 6), experimenter error (n = 1), seeing our stimuli previously (n = 1), or for napping between phases in the Wake condition (n = 37). Participants were not instructed to refrain from napping to ensure the honest reporting of any napping behavior. Additionally, although we excluded participants who slept less than 4 hours or who did not turn in a sleep diary, including these participants does not change the main finding on the recognition lineup test, χ^2^(2, *N =* 103) = 1.78, *p =* .41.

**Additional Results**

***Sleepiness and Mood:*** Subjective sleepiness [1] was assessed during the Encoding and Test phases. A repeated measures ANOVA with Phase (Encoding, Test) as the within-subjects factor and Condition (Wake, Sleep) as the between-subjects factor showed a main effect of Condition such that the Sleep group reported higher sleepiness than the Wake group across both Phases, *F*(1, 86) = 14.36, *MSE =* 1.47, *p* < .001 (Table S1). There was no main effect of Phase, *F*(1, 86) = .36, *MSE* = 1.31, *p* = .55, and no interaction, *F*(1, 86) = 3.49, *MSE =* 1.31, *p =* .07. For the control groups, we averaged the two SSS scores from the Encoding and Test phases since no delayed retention interval separated the phases. An independent samples t-test showed no difference in sleepiness between AM and PM, *t*(108) = .06, *p =* .95 (Table S1).

Positive and negative affect [2] was assessed during both the Encoding and Test phases. For the experimental groups, we ran two repeated measures ANOVAs, one for the positive affect score and another for the negative affect score, with Phase (Encoding, Test) as the within-subjects factor and Condition (Wake, Sleep) as the between-subjects factor. For positive affect, there was no main effect for Phase, *F*(1, 86) = 1.47, *MSE =* 20.26, *p =* .23, or Condition, *F*(1, 86) = 1.55, *MSE =* 90.74, *p =* .22. There was a significant interaction, *F*(1, 86) = 12.90, *MSE =* 20.26, *p =* .001, indicating that the Wake group rated themselves as having higher positive affect than Sleep during the Test phase, *t*(86) = 2.53, *p =* .01, but not the Encoding phase, *t*(86) = -.43, *p =* .67 (Table S1).

For negative affect, there was a significant main effect of Phase, *F*(1, 86) = 4.44, *MSE =* 4.34, *p =* .04, indicating that participants rated themselves as having more negative affect during the Encoding phase. There was also a significant main effect of Condition with the Sleep group rating themselves as having more negative affect than Wake, *F*(1, 86) = 5.25, *MSE =* 15.83, *p =* .02. However, this main effect of Condition is qualified by a significant interaction, *F*(1, 86) = 5.33, *MSE =* 4.34, *p =* .02, which revealed that the Sleep group rated themselves as having more negative affect than Wake only during the Encoding phase, *t*(86) = -2.93, *p =* .004, but not the Test phase, *t*(86) = -1.03, *p =* .31 (Table S1).

For the control groups, we averaged the two positive affect scores together and the two negative affect scores together since no delayed retention interval separated these phases. Independent samples t-tests showed no difference in positive affect between AM and PM, *t*(108) = .77, *p =* .75, but a significant effect of negative affect, *t*(108) = -3.46, *p =* .001, with the AM group reporting more negative affect than the PM group (Table S1). Despite this difference in affect, the groups performed equally well at identifying the perpetrator from the lineup.

Since we found group differences in sleepiness and mood in the experimental groups, we added them to a model of eyewitness identifications to see if they affected performance. We ran a forward stepwise multinomial logistic regression with Condition (Wake, Sleep), sleepiness during Encoding and Test, positive affect during Encoding and Test, and negative affect during Encoding and Test as possible predictors of eyewitness identifications. As predicted, none of these variables were significant predictors of performance and none were added to the model in this forward stepwise procedure. Therefore, even though we found group differences in sleepiness and mood, these factors do not appear to affect eyewitness identifications.

Table S1. Sleepiness and Affect Scores in Experiment 1.

|  | **Encoding Phase** | | | **Test Phase** | | |
| --- | --- | --- | --- | --- | --- | --- |
|  | **Sleepiness** | **Positive Affect** | **Negative Affect** | **Sleepiness** | **Positive Affect** | **Negative Affect** |
| **Wake** | 2.87 (.80) | 24.45 (7.06) | 12.38 (2.67) | 2.45 (1.10) | 26.06 (7.57) | 12.45 (2.68) |
| **Sleep** | 3.24 (1.22) | 25.10 (7.02) | 14.49 (4.02) | 3.46 (1.53) | 21.83 (8.13) | 13.10 (3.28) |
| **AM** | 2.80 (1.10) | 25.18 (9.23) | 12.26 (2.58) | 2.86 (1.10) | 25.26 (7.97) | 12.18 (1.97) |
| **PM** | 2.98 (1.15) | 23.83 (8.58) | 14.76 (4.89) | 2.66 (1.21) | 25.64 (7.83) | 14.46 (4.67) |

Self-reported sleepiness and positive and negative affect scores with standard deviations (in parentheses) for the experimental (Wake, Sleep) and control (AM, PM) groups during the Encoding and Test phases of Experiment 1. Higher numbers indicate higher sleepiness and higher positive or negative affect.

***Operation Span:*** OSPAN [3] was used to asses working memory capacity. For each participant, we calculated an OSPAN score which reflects the total number of letters accurately recalled regardless of order. We used a two factor between-subjects ANOVA with Condition (Wake, Sleep) and Identification category (Correct, False, No Identification) as factors. There was no main effect of Condition, *F*(1, 82) = 1.17, *MSE* = 125.39, *p* = .28, no main effect of Identification category, *F*(2, 82) = .01, *MSE* = 125.39, *p =* .99, and no interaction, *F*(2, 82) = .77, *MSE* = 125.39, *p* = .47 (Table S2). For the control groups, we used a two factor between-subjects ANOVA with Test Time (AM, PM) and Identification category as factors. There was no effect of Test Time, *F*(1, 104) = 3.25, *MSE* = 174.24, *p =* .07, no main effect of Identification category, *F*(2, 104) = .10, *MSE* = 174.24, *p =* .90, and no interaction, *F*(2, 104) = .53, *MSE* = 174.24, *p =* .59 (Table S2).

Table S2. Operation Span Performance for Experiment 1.

|  | **Correct ID** | **False ID** | **No ID** |
| --- | --- | --- | --- |
| **Wake** | 58.58 (10.24) | 61.91 (10.95) | 57.58 (11.63) |
| **Sleep** | 57.15 (11.23) | 54.58 (10.63) | 58.11 (13.91) |
| **AM** | 59.88 (11.43) | 58.00 (11.78) | 58.00 (7.72) |
| **PM** | 50.59 (16.93) | 55.89 (9.74) | 52.54 (10.94) |

Mean Total OSPAN score and standard deviations (in parentheses) for the experimental (Wake, Sleep) and control (AM, PM) groups in Experiment 1.

***Chronotype****:* We assessed chronotype with the Morning-Eveningness Questionnaire [4]. The majority of participants indicated no strong diurnal preference and were categorized as “Neither Morning nor Evening” types. The next largest category was “Moderate Evening.” Very few participants were categorized as “Definite Evening,” “Moderate Morning,” or “Definite Morning” (Table S3). We were mainly interested if evening-type individuals (n = 27, collapsed across the “Definite Evening” and “Moderate Evening” chronotypes), performed differently based on time-of-day. Chi-Square analyses showed that evening-type individuals did not perform differently based on time-of-day for either the experimental, χ^2^ (2, *N* = 27) = 2.25, *p* = .32, or control groups, χ^2^ (2, *N* = 30) = 1.34, *p* = .51. We could not perform this same analysis for morning-type individuals due to the small number of participants in the “Definite Morning” and “Moderate Morning” categories.

Table S3. Time-of-Day Preferences in Experiment 1.

| **Classification** | **Wake** | **Sleep** | **AM** | **PM** |
| --- | --- | --- | --- | --- |
| Definite Morning | 0% (0) | 0% (0) | 2% (1) | 0% (0) |
| Moderate Morning | 2% (1) | 5% (2) | 10% (5) | 3% (2) |
| Neither Morning nor Evening | .70 (33) | .61 (25) | 61% (31) | 73% (43) |
| Moderate Evening | .28 (13) | .32 (13) | 24% (12) | 20% (12) |
| Definite Evening | 0 (0) | .02 (1) | 4% (2) | 3% (2) |

Percentage and total number (in parentheses) of participants in each chronotype category, assessed using the Morningness-Eveningness Questionnaire, for the experimental (Wake, Sleep) and control (AM, PM) groups in Experiment 1.

***Sleep*** ***quality****:* Overall sleep quality was assessed using the Pittsburgh Sleep Quality Index (PSQI) [5]. Invalid answers from 2 participants in the experimental groups and 1 participant in the control groups prevented calculating a PSQI score and are thus excluded from the analyses. An independent samples t-test showed no differences in sleep quality between Wake (*M* = 5.42, *SD =* 2.59) and Sleep (*M* = 5.66, *SD* = 2.10), *t*(84) = -.46, *p* = .65. There were also no differences in PSQI scores between the AM (*M =* 5.37, *SD* = 2.42) and PM (*M =* 6.05, *SD* = 3.35) control groups, *t*(107) = -1.20, *p* = .23.

***Sleep diaries****:* Participants used sleep diaries to track their sleeping habits for up to one week prior to the experiment. Some participants in the experimental (n = 20) and control (n = 14) groups turned in partially completed sleep diaries for the week leading up to the experiment (e.g. completed only 5 of the 7 nights). For these participants, we used the data that was available and averaged based on the number of days completed. We performed independent sample t-tests to determine if there were any differences in average weekly sleep duration or average sleep duration the night before the Encoding phase. For the experimental groups, we found no differences in average amount slept the week prior to the experiment between Wake (*M =* 7hrs 47min, *SD =* 40min) and Sleep (*M =* 7hrs 55min, *SD =* 1hr 4min), *t*(86) = -.69, *p* = .49, or average amount of sleep the night before Encoding between Wake (*M =* 7hrs 29min, *SD =* 1hr 33min) and Sleep (*M* = 7hrs 32min, *SD* = 1hrs 56min), *t*(86) = -.11, *p* = .91. For the control groups, there were also no differences in average weekly sleep duration between AM (*M =* 7hrs 43min, *SD* = 45min) and PM (*M =* 7hrs 59min, *SD =* 53min), *t*(108) = -1.72, *p* = .09, or sleep duration the night before encoding between AM (*M* = 7hrs 56min, *SD* = 1hr 38min) and PM (*M =* 7hrs 58min, *SD =* 1hr 25min), *t*(108) = -.08, *p* = .93.

**Experiment 2**

**Additional Methods**

**Participants.** Participants were excluded for: incomplete datasets (n = 23), reporting a sleep disorder (n = 7), sleeping less than 4 hours before the encoding or test phases (n = 4), previously watching the 60 Minutes special (n = 2), experimenter error (n = 1), failing to watch the mock-crime video (n = 2), or for napping between phases in the Wake group (n = 50). Similar to Experiment 1, participants were not instructed to refrain from napping and including the participants who slept less than 4 hours or who did not turn in a sleep diary did not change the main finding on the recognition lineup test, χ^2^(1, *N =* 111) = 7.46, *p =* .01.

**Additional Results**

***Sleepiness and Mood****:* We assessed sleepiness [1] using a two factor repeated measures ANOVA with Phase (Encoding, Test) as the within-subjects factor and Condition (Wake, Sleep) as the between-subjects factor. There was no main effect of Phase, *F*(1, 94) = 1.18, *MSE* = .94, *p* = .28, but we found a significant main effect of Condition, *F*(1, 94) = 5.98, *MSE* = 1.63, *p* = .02, that was qualified by a significant Phase X Condition interaction, *F*(1, 94) = 22.24, *MSE* = .94, *p<*.001, indicating that the Sleep group rated themselves as more sleepy than the Wake group during the Test phase, *t*(94) = -4.49, *p<*.001, but not during the Encoding phase, *t*(94) = .97, *p* = .33 (Table S4). For the control groups, we averaged the two SSS scores from the Encoding and Test phases since participants completed both in a single session. An independent samples t-test showed no difference in self-reported sleepiness between AM and PM, *t*(140) = .39, *p =* .70 (Table S4).

Positive and negative affect [2] was assessed during both the Encoding and Test phases. For the experimental groups, we performed two repeated measures ANOVAs, one for positive affect and one for negative affect, with Phase (Encoding, Test) as the within-subjects factor and Condition (Wake, Sleep) as the between-subjects factor. For positive affect, there was not a significant main effect of Phase, *F*(1, 94) = 2.63, *MSE =* 19.88, *p =* .11, or Condition, *F*(1, 94) = 1.46, *MSE =* 80.72, *p =* .23, but there was a significant interaction, *F*(1, 94) = 36.89, *MSE =* 32.20, *p <* .001, indicating that the Wake group reported higher positive affect during the Encoding phase, *t*(94) = 3.53, *p =* .001 (Table S4). This is consistent with Experiment 1 in which the Wake group also reported more positive affect during the Encoding phase.

For negative affect, there were also no significant main effects for Phase, *F*(1, 94) = 3.40, *MSE =* 4.82, *p =* .07, or Condition, *F*(1, 94) = 2.37, *MSE =* 17.74, *p =* .13. There was a significant interaction, *F*(1, 94) = 5.37, *MSE =* 4.82, *p =* .02, indicating that the Wake group reported higher negative affect than the Sleep group during the Encoding phase, *t*(94) = 2.44, *p =* .02 (Table S4). Therefore, the Wake group reported higher positive and negative affect during the Encoding phase.

For the control groups, we averaged the two positive affect scores together and the two negative affect scores together collected during the Encoding and Test phases since participants since both phases in a single session. Independent sample t-tests showed that AM and PM did not differ in positive, *t*(140 ) = -.81, *p =* .42, or negative affect, *t*(140) = -1.57, *p =* .12 (Table S4).

Given that performance differed on the recognition lineup test and that we found group differences in sleepiness and mood during the Test phase, we tested whether sleepiness or mood predicted performance on the lineup test above that of Condition. We ran a forward conditional binary logistic regression with Condition (Wake, Sleep), sleepiness during the Encoding and Test phases, positive affect during the Encoding and Test phases, and negative affect during the Encoding and Test phases as possible predictors. As expected, only Condition was a significant predictor of eyewitness identifications (β = .99, *p* = .02), and therefore added to the model in this forward conditional procedure.

Table S4. Sleepiness and Affect Scores in Experiment 2.

|  | **Encoding Phase** | | | **Test Phase** | | |
| --- | --- | --- | --- | --- | --- | --- |
|  | **Sleepiness** | **Positive Affect** | **Negative Affect** | **Sleepiness** | **Positive Affect** | **Negative Affect** |
| **Wake** | 3.00 (.98) | 23.51 (7.04) | 13.53 (3.15) | 2.49 (1.20) | 26.13 (7.62) | 13.68 (3.94) |
| **Sleep** | 2.79 (1.12) | 25.61 (6.79) | 13.33 (3.62) | 3.61 (1.22) | 20.88 (6.78) | 12.00 (2.43) |
| **AM** | 2.88 (1.15) | 23.63 (7.86) | 12.66 (3.70) | 3.01 (1.12) | 23.34 (7.34) | 13.26 (3.78) |
| **PM** | 2.81 (1.19) | 24.45 (6.98) | 13.80 (3.55) | 2.94 (1.19) | 24.36 (7.05) | 13.88 (3.76) |

Self-reported sleepiness and positive and negative affect scores with standard deviations (in parentheses) for the experimental (Wake, Sleep) and control (AM, PM) groups during the Encoding and Test phases of Experiment 2.

***Operation Span****:* Operation Span (OSPAN) [3] was used assess working memory capacity. For the experimental groups, we used a two factor between-subjects ANOVA with Condition and Identification category as factors. There was no main effect of Condition, *F*(1, 92) = .11, *MSE* = 126.27, *p* = .74, no main effect of Identification category, *F*(1, 92) = .01, *MSE* = 126.27, *p* = .94, and no interaction, *F*(1, 92) = .26, *MSE* = 126.27, *p* = .61 (Table S5). For the control groups, we used a two factor between-subjects ANOVA with Test Time and Identification category as factors. There was no main effect of Test Time, *F*(1, 138) = 3.26, *MSE* = 186.44, *p* = .07. However, there was a main effect of Identification category; individuals who made a No Identification had higher OSPAN scores than those who made a False Identification, *F*(1, 138) = 4.57, *MSE* = 186.44, *p* = .03. There was no interaction between Test Time and Identification category *F*(1, 138) = 1.72, *MSE* = 186.44, *p* = .19. (Table S5).

Table S5. Operation Span Performance for Experiment 2.

|  | **False ID** | **No ID** |
| --- | --- | --- |
| **Wake** | 57.86 (12.36) | 56.83 (8.28) |
| **Sleep** | 57.44 (13.55) | 58.84 (9.44) |
| **AM** | 50.06 (18.08) | 58.10 (13.69) |
| **PM** | 57.32 (13.11) | 59.24 (9.49) |

Mean OSPAN score and standard deviations (in parentheses) for the experimental (Wake, Sleep) and control groups (AM, PM) in Experiment 2.

***Chronotype****:* The Morningness-Eveningness Questionnaire (MEQ) [4] was used to assess time-of-day preference. The pattern of results in the MEQ in this experiment was quite similar to Experiment 1 – the vast majority of participants were categorized as “Neither Morning nor Evening” (Table S6). For both the experimental and control groups, we were mainly interested if evening oriented participants’ performance varied based on time-of-day. For these analyses, we collapsed across the “Definite Evening” and “Moderate Evening” categories in the experimental (n = 34) and control (n = 42) groups. For the experimental groups, a Chi-Square analysis was not significant, indicating that evening-type individuals did not perform differently based on time-of-day, χ^2^ (1, *N* = 34) = 3.03, *p* = .08. For the control groups, the Chi-Square analysis showed no difference in performance due to time-of-day, χ^2^ (1, *N* = 42) = .10, *p* = .76. Due to the small number of participants in the “Definite Morning” and “Moderate Morning” chronotypes, the same analyses could not be completed for morning-type individuals.

Table S6. Time-of-Day Preferences in Experiment 2.

| **Classification** | **Wake** | **Sleep** | **AM** | **PM** |
| --- | --- | --- | --- | --- |
| Definite Morning | 0% (0) | 0% (0) | 0% (0) | 0% (0) |
| Moderate Morning | 6% (3) | 5% (2) | 6% (4) | 4% (3) |
| Neither Morning nor Evening | 64% (34) | 54% (23) | 67% (49) | 65% (45) |
| Moderate Evening | 28% (15) | 40% (17) | 25% (18) | 28% (19) |
| Definite Evening | 2% (1) | 2% (1) | 3% (2) | 3% (2) |

Proportion and total number (in parentheses) of participants in each chronotype category, assessed using the Morningness-Eveningness Questionnaire, in the experimental (Wake, Sleep) and control (AM, PM) groups for Experiment 2.

***Sleep Quality****:* The Pittsburgh Sleep Quality Index (PSQI) [5] was used to measure overall sleep quality. Invalid answers from 3 participants in the experimental groups and 4 participants in the control groups prevented calculating a PSQI score and are therefore excluded from the analyses. Two independent samples t-tests showed no differences in sleep quality between Sleep (*M =* 5.07, *SD =* 2.21) and Wake (*M =* 5.51, *SD* = 2.02), *t*(92) = 1.00, *p* = .32, or between AM (*M* = 6.01, *SD* = 2.89) and PM (*M =* 5.80, *SD* = 2.73), *t*(136) = .45, *p* = .65.

***Sleep Diaries****:* Participants tracked their sleeping habits for up to one week prior to the experiment. For participants in the experimental (n = 19) and control (n = 14) groups who turned in partially completed sleep diaries (e.g. 5 of the 7 nights), we used the available data and averaged over the number of days completed. Similar to Experiment 1, we found no difference in average weekly sleep duration between Wake (*M =* 7hrs 40min, *SD =* 50min) and Sleep (*M* = 7hrs 52min, *SD =* 44min), *t*(94) = -1.25, *p* = .21, or average amount slept the night before encoding between Wake (*M =* 7hrs 7min, *SD* = 1hr 20min) and Sleep (*M* = 7hrs 14min, *SD* = 1hr 31min), *t*(94) = -.36, *p* = .72. These analyses suggest that the high False Identification rate in the Wake group is not a product of less sleep. In the control groups, we again found no differences in average weekly sleep duration between AM (*M =* 7hrs 52min, *SD =* 49min) and PM (*M* = 7hrs 47min, *SD =* 59min), *t*(140) = .51, *p* = .61, or average sleep duration the night before encoding between AM (*M =* 8hrs 10min, *S*  = 1hr 42min) and PM (*M* = 7hrs 53min, *SD =* 1hr 39min), *t*(140) = .96, *p* = .34.

**References**

1. Hoddes, E., Zarcone, V., Smythe, H., Phillips, R., & Dement, W.C. (1973). Quantification of sleepiness: a new approach. *Psychophysiology*, *10*(4), 431-436.
2. Watson, D., Clark, L.A., & Tellegen, A. (1988). Development and validation of brief measures of positive and negative affect: the PANAS scales. *Journal of personality and social psychology*, *54*(6), 1063.
3. Unsworth, N., Heitz, R.P., Schrock, J.C., & Engle, R.W. (2005). An automated version of the operation span task. Behavior Research Methods, 37(3), 498 –505. doi:10.3758/BF03192720.
4. Horne, J.A., & Ostberg, O. (1975). A self-assessment questionnaire to determine morningness-eveningness in human circadian rhythms. *International journal of chronobiology*, *4*(2), 97-110.
5. Buysse, D.J., Reynolds, C.F., Monk, T.H., Berman, S.R., & Kupfer, D.J. (1989). The Pittsburgh Sleep Quality Index (PSQI): A new instrument for psychiatric research and practice. *Psychiatry Research*, *28*(2), 193-213.
